# Induced membrane technique versus one-stage autografting in management of atrophic nonunion of long bone in the lower limb: clinical and health burden outcomes

**DOI:** 10.1186/s13018-023-04296-1

**Published:** 2023-11-09

**Authors:** Hu Zhang, Jingshu Fu, Shen Jie, Xiaohua Wang, Shulin Wang, Hongri Wu, Yongjun Hu, Chunji Huang

**Affiliations:** 1 Army Medical University (Third Military Medical University), Gaotanyan Street No.30, Shangpingba District, Chongqing, 400038 People’s Republic of China; 2 Banan Hospital of Chongqing Medical University, Banan District, Longzhouwan Street No. 659, Chongqing City, 400038 People’s Republic of China; 3 Department of Orthopaedics, Southwest Hospital, Army Medical University (Third Military Medical University), Gaotanyan Street No.30, Shangpingba District, Chongqing, 400038 People’s Republic of China; 4 Department of Orthopedics, Navy 905th Hospital, Naval Medical University, Shanghai, People’s Republic of China

**Keywords:** Nonunion, Induced membrane technique, Autologous bone grafting, Diamond concept

## Abstract

**Objective:**

In this study, we aimed to compare the outcomes of the two-stage induced membrane technique (IMT) and one-stage autografting in the treatment of aseptic atrophic nonunion in lower limb long bones.

**Methods:**

From January 2014 to January 2022, we reviewed all surgically treated long bone nonunion patients, including patients aged 18 years or older with atrophic nonunion, who were either treated with the two-stage induced membrane technique (IMT) or one-stage autografting. Outcome parameters interns of clinical, quality of life and healthcare burden were recorded and retrospectively analysed between the two treatment populations. The follow-up time was at least 1 year.

**Results:**

In total, 103 patients who met the criteria for aseptic atrophic nonunion were enrolled. Among them, 41 (39.8%) patients were treated with two-stage IMT, and 62 (60.2%) patients were treated with one-stage autologous bone grafting. The follow-up time was 12 to 68 months, with an average of 28.4 months. The bone healing rate was comparable in both groups (IMT: 92.7% vs. one-stage grafting: 91.9%, *P* = 0.089) at 12 months post-operation, and the bone healing Lane–Sandhu score was superior in the IMT group (mean: 8.68 vs. 7.81, *P* = 0.002). Meanwhile, the SF-12 scores of subjective physical component score (PCS) (mean: 21.36 vs. 49.64, *P* < 0.01) and mental health component score (MCS) (mean: 24.85 vs. 46.14, *P* < 0.01) significantly increased in the IMT group, as well as in the one-stage grafting group, and no statistically significant difference was found within groups. However, the total hospital stays (median: 8 days vs. 14 days, *P* < 0.01) and direct medical healthcare costs (median: ¥30,432 vs. ¥56,327, *P* < 0.05) were greater in the IMT group, while the complications (nonunion 8, infection 3, material failure 2, and donor site pain 6) were not significantly different between the two groups (17.1% vs. 19.4, *P* = 0.770).

**Conclusion:**

The data indicate that two-stage method of IMT serves as an alternative method in treating atrophic nonunion; however, it may not be a preferred option, in comprehensive considering patient clinical outcomes and healthcare burden. More evidence-based research is needed to further guide clinical decision-making.

## Introduction

Despite ongoing advances in health care increasing life expectancy, trauma has occurred, and skeletal failure to heal remains a continuing problem regardless of patient age or activity level. The literature [1, 2] shows that the incidence of nonunion in long bone fractures generally varies from 5 to 10%, and it is sometimes as high as 42.7% in high-risk patients. Nonunion often presents as pain or abnormal activity at the fracture site, with serious negative effects on both patient health and quality of life [3, 4]. The current treatment principles of nonunion are mainly based on the “diamond” theory [1, 5, 6]. This focuses on improving the peripheral blood supply, osteoinductive factors, osteoblasts, bone conduction matrix and mechanical stability. The variation in choosing different treatments was mainly according to the underlying causes or clinical types of nonunion.

Among all nonunion types, atrophic nonunion [1, 7–9] is common in clinical practice. It usually lacks local blood supply or low osteogenic potential, thus resulting in less callus formation on imaging. Of note, although these patients do not show any signs of infection, the low-toxicity infection is also a possible cause of atrophic nonunion. Based on the "diamond theory" [1, 6], such intervention strategies for nonunion should have the main goal of improving the biological environment of the fracture site. These include the treatment of potential low-toxicity infections [9]. The traditional standard intervention is local debridement plus autologous cancellous bone grafting. In addition, the recent two-stage induced membrane technique (IMT) [10–12] is also a treatment option. The first phase of IMT is to insert polymethylmethacrylate (PMMA) into the nonunion gap and form a bioactive membrane (induced membrane, IM) around the PMMA. In the second stage (6–8 weeks), the bone cement is removed, and cancellous bone grafting is performed. IM has high vascularization and can secrete various growth factors [10, 13, 14]. These include transforming growth factor beta 1 (TGF-β1), fibroblast growth factor 2 (FGF-2), bone morphogenetic protein 2 (BMP-2) and vascular endothelial growth factor (VEGF); IM can also mobilize osteogenic precursor cells, thus favouring bone healing. Moreover, bone cement can be used to add antibiotics to provide local anti-infection treatment [15].

However, it is unclear whether the stage method of IMT should be a priority treatment option in clinical decision-making compared to one-stage autograft. Based on the “diamond” theory, we speculate that two-stage IMT treatment can achieve better results in the management of atrophic nonunion by further improving the local biological environment. Therefore, we performed a retrospective cohort study and aimed to compare the outcome of two-stage IMT treatment versus one-stage autograft in the management of aseptic atrophic nonunion in lower limb long bones. In addition to related clinical results, patients’ quality of life and healthcare burden were also investigated and compared between these two methods. Our results may be helpful for clinical decision-making both by surgeons and patients in the management of atrophic nonunion.

## Patients and methods

This is a retrospective cohort study. From January 2014 to January 2022, we reviewed all nonunion patients surgically treated at our clinic centre. The diagnosis of nonunion was made according to the U.S. Food and Drug Administration (FDA) criteria [1, 6, 16]: patients were at least 9 months after fracture and showed no signs of further healing within three consecutive months. Atrophic nonunions [9, 17] were defined as previous fractures that exhibited a lack of healing with no callus formation on X-ray. Patients who met the following criteria were included: long bone of the lower limb; atrophic nonunion; age 18 years old or greater; and received either two-stage IMT treatment or one-stage autologous bone grafting. The exclusion criteria were as follows: patients with coinfection according to preoperative clinical or imaging signs; patients with cancer; and patients with incomplete follow-up data or a follow-up time of less than 1 year. This retrospective study was approved by the local medical ethics committee.

### Surgical procedures

Before surgical treatment, the previous surgery, clinical types of bone nonunion (atrophic or hypertrophic, based on the local biological microenvironment), the possible size of the defect after debridement must be carefully assessed, as well as the possibility of current or previous infection. The alignment of the bone and the extremity must also be considered. Of note, both IMT treatment and one-stage autologous bone grafts are currently acceptable treatment methods. In clinical practice, patients voluntarily select IMT treatment or one-stage autologous bone grafting, although the final surgery is also at the discretion of the responsible surgeon.

**Fig. 1:**
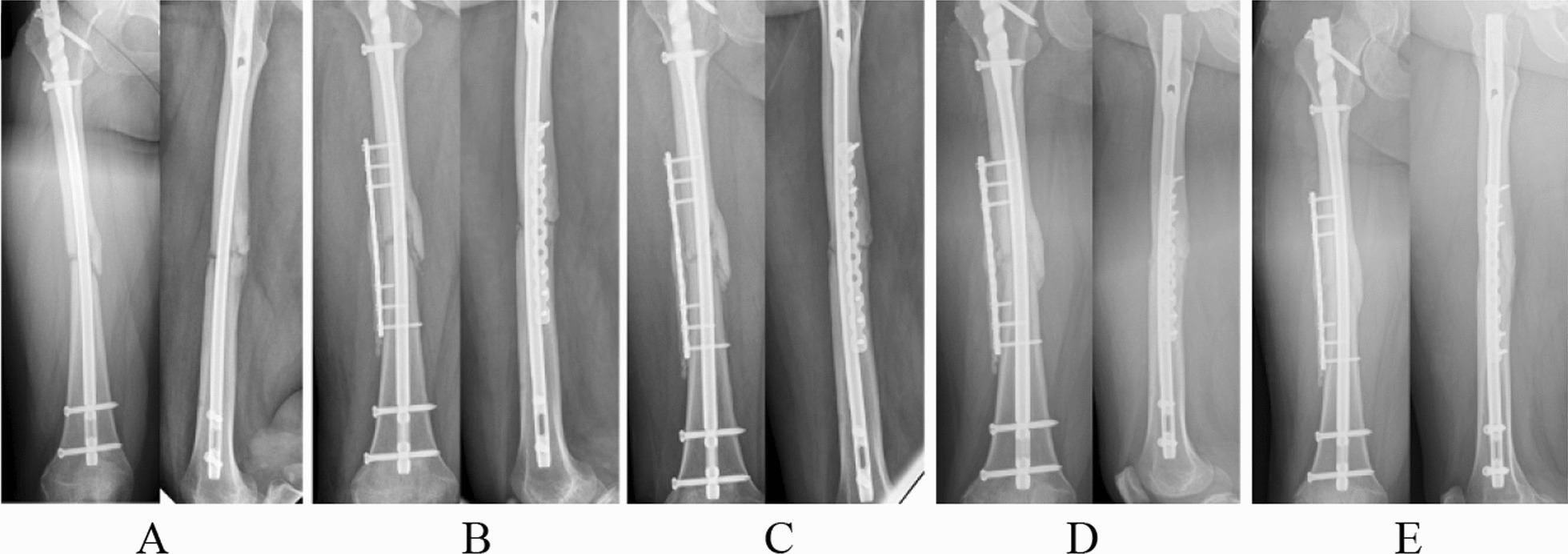
A female patient (25 years old) with bone nonunion in the right femur was treated with one-stage autologous bone grafting and had an open fracture before; no bacteria were detected after surgery. **A** Anteroposterior and lateral radiographs showed a clear fracture line and less callus formation at the fracture site after 12 months of initial osteosynthesis; **B**–**E** radiographs at 2, 6, 12, and 18 months after one-stage autologous bone grafting treatment. Bone union was achieved at 12 months, and complete bone consolidation was achieved at 18 months

For patients treated with one-stage autologous bone grafting, the main surgical procedures included debridement and immediate bone grafting. First, the surrounding poor-quality tissue or newborn bone is extensively excised until fresh bleeding healthy tissue is left. Of note, whether a prior surgical implant was removed is determined according to whether fixation was ineffective or suspected of underlying low-toxicity infection [17]. Second, the nonunion is stabilized with suitable biomechanical implants accordingly. The definite fixation includes an intramedullary nail, osteosynthesis plate and intramedullary nail plus plate. Finally, the bone gap or defect is filled with autologous bone grafts (Fig. 1), which are obtained from the anterior superior iliac. During each procedure, multiple (often 3–5) tissue samples are collected and analysed for any type of microbial infection.

**Fig. 2:**
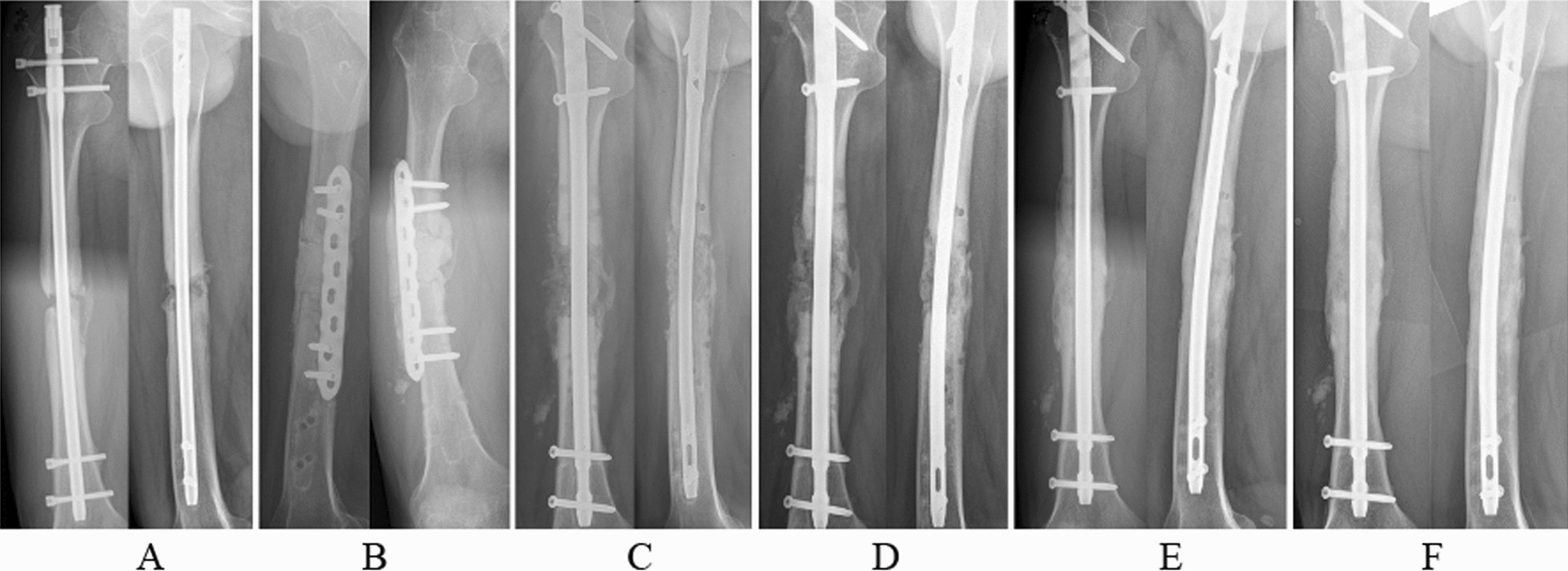
A male patient (28 years old) with bone nonunion in the right femur was treated with the staged method of the induced membrane technique (IMT), and a low-toxicity infection (coagulase-negative Staphylococcus spp.) was detected after surgery. **A** Preoperative radiographs showed less callus formation at the fracture site after initial osteosynthesis. **B** Debridement and bone gap filled with antibiotic bone cement. **C**–**F** Radiographs at 2, 6, 12, and 18 months after IMT treatment. Bone union was achieved at 12 months, and complete bone consolidation was achieved at 18 months

In the two-stage method of IMT therapy, there are usually two separate surgical steps. In the first step, radical debridement of the nonhealing bone end and surrounding soft tissue is performed, and then the disconnected ends are filled with polymethylmethacrylate (PMMA) spacers. All PMMA is impregnated with antibiotics (each 40 g of PMMA powder is mixed with 4 g of vancomycin powder and 500 mg of gentamicin [18]) to treat the underlying infection. In the second step (after 6–8 weeks), the spacer is removed with careful protection of the inducing membrane, and then autologous bone grafting is performed (Fig. 2) with bone also obtained from the anterior superior iliac. In all other respects, the second step of IMT treatment is identical to one-stage autologous bone grafting.

### Follow-up and outcome measures

To evaluate clinical bone healing outcomes and complications, both groups (two-stage IMT treatment versus one-stage autograft treatment) had regular outpatient visits at 2, 6, 9 and 12 months after surgery, and X-ray images of the involved bone were routinely performed to provide continuous observation of fracture healing. The primary outcome was fracture healing. Bone union was defined as an image showing continuous callus formation in three bone cortices. The additional Lane and Sandhu score [19, 20] was used to further compare bone healing between the two groups.

In addition, quality of life evaluated by the 12-item Health Report Brief Form (SF-12) [3, 4] was also collected pre- and postoperatively. Patient follow-up was conducted by full-time clinical research specialists, and complications were also recorded. Meanwhile, the two independent and experienced trauma surgeons were responsible for the clinical assessment of mechanical stability, weight-bearing, postoperative pain, and signs of infection. In this retrospective study, data regarding treatment burden with respect to total hospital stays and direct healthcare costs were collected in addition to information related to treatment and clinical outcome and patient demographics.

### Statistical analysis

Statistical analysis was performed using SPSS 22.0 software, and the Levene test was used to determine whether measurement data were normally distributed. Continuous variables were expressed as ± *s*, and a *t* test with independent samples was used to compare two groups. Count data were expressed as a rate and were compared by the *χ*^2^ test. Data that did not follow the distribution of variance were analysed with a nonparametric test. In the two-tailed test, the differences between the two groups were considered statistically significant when the *P* value was < 0.05.

## Results

### Demographic characteristics

According to the study criteria, 103 patients with atrophic nonunion were enrolled, including 70 femurs and 33 tibias. All these patients completed at least a 1-year postoperative follow-up ranging from 12 to 68 months, with a mean of 28.4 months. According to the surgical methods, 41 patients were treated with two-stage IMT, and 62 patients were treated with one-stage autologous bone grafting. Among them, patients treated with IMT included 24 femurs and 17 tibias, with a mean age of 39.42 ± 11.90 years. On the other hand, the one-stage autologous grafting group included 46 femurs and 16 tibias with a mean age of 39.44 ± 10.39 years. The basic demographic characteristics of gender, age, smoking and diabetes mellitus, prior trauma and osteosynthesis are presented in Table 1, showing no significant difference between the two groups.

**Table 1:** Baseline characteristics of 103 patients with atrophic nonunion

Variable	Total (*N* = 103)	Patients treated with two-stage IMT (*N* = 41)	Patients treated with one-stage autograft (*N* = 62)	*P* value
Mean age (years)	39.43 ± 11.27	39.42 ± 11.90	39.44 ± 10.39	0.993
Age stratification				
< 40 years	46 (44.7)	19 (46.3)	27 (43.5)	0.841
≥ 40 years	57 (55.3)	22 (53.7)	35 (56.5)	
Gender				
Male	79 (76.7)	30 (73.2)	49 (79.0)	
Female	24 (23.3)	11 (26.8)	13 (21.0)	
BMI (kg/m^2^)	24.6 ± 1.8	24.9 ± 1.9	24.2 ± 1.8	0.247
Site				
Tibia	33 (32.0)	17 (41.5)	16 (25.8)	0.131
Femur	70 (68.0)	24 (58.5)	46 (74.2)	
Prior fracture				
Open	67 (65.0)	30 (73.2)	37 (59.7)	0.206
Closed	36 (35.0)	11 (26.8)	25 (40.3)	
Tobacco use				
Yes	32 (31.1)	12 (29.3)	20 (32.3)	0.829
No	71 (68.9)	29 (70.7)	42 (67.7)	
Diabetes				
Yes	13 (12.6)	6 (14.6)	7 (11.3)	0.763
No	90 (87.4)	35 (85.4)	55 (88.7)	
Initial osteosynthesis				
Intramedullary nail	42 (40.0)	17 (41.5)	25 (40.3)	0.935
Osteosynthesis plate	35 (33.3)	13 (31.7)	22 (35.5)	
Others^a^	26 (24.7)	11 (26.8)	15 (24.2)	

^a^External fixation, wire or conservative

### Treatment parameters and clinical outcome

Of all 103 patients, 39 (37.9%) chose an intramedullary nail for definite fixation, 26 (25.2%) chose an osteosynthesis plate alone, and 38 (36.9%) chose an intramedullary nail plus plate. Moreover, a mean bone defect gap of 2.00 ± 0.46 occurred in all patients after debridement. Intraoperative culture and PCR tests also demonstrated that a proportion of atrophic nonunion patients (n = 17, 16.5%) had low toxicity infections, among which coagulase-negative *Staphylococcus* spp. (*n* = 8, 7.77%) were the most common bacteria. The fixation methods (*P* = 0.603), bone defect gaps (*P* = 0.077) and detected species (*P* = 0.790) were not significantly different between the patients with two-stage IMT treatment and the patients with one-stage autologous grafts (Tables 2, 3).

**Table 2:** Treatment and outcome of 103 patients with atrophic nonunion

Variable	Total (*N* = 103)	Patients treated with two-stage IMT (*N* = 41)	Patients treated with one-stage autograft (*N* = 62)	*P* value
				One-stage group versus two-stage group
Definite fixation				
Intramedullary nail	39 (37.9)	13 (31.7)	26 (41.9)	0.603
Osteosynthesis plate	26 (25.2)	11 (26.8)	15 (24.2)	
Intramedullary nail + plate	38 (36.9)	17 (41.5)	21 (33.9)	
Bone defect gap, cm	2.00 ± 0.46	2.11 ± 0.56	1.93 ± 0.36	0.077
Healthcare burden				
Total hospital stay, median (range) days	12 (7–32)	8 (7–18)	14 (10–32)	< 0.01
Total direct healthcare costs, ¥	46,480 (23,467–98,792)	30,432 (23,467–67,618)	56,327 (46,063–98792)	0.026
Bone consolidation				
Union, yes	95 (92.2)	38 (92.7)	57 (91.9)	0.089
Lane Sandhu scores	8.16 ± 1.55	8.68 ± 0.96	7.81 ± 1.76	0.002
Complications	19 (18.4)	7 (17.1)	12 (19.4)	0.770
Nonunion	8 (7.8)	3 (7.3)	5 (8.1)	
Infection	3 (2.9)	1 (2.4)	2 (3.2)	
Material failure	2 (6.9)	1 (2.4)	1 (1.6)	
Donor site pain	6 (5.8)	2 (4.9)	4 (6.5)	
SF-12 PCS				
Preoperative	22.16 ± 6.84	21.36 ± 5.96	22.90 ± 7.59	0.383
1 year post-op	49.94 ± 4.08	49.64 ± 3.75	50.23 ± 4.41	0.580
Significance within group		< 0.01	< 0.01	
SF-12 MCS				
Preoperative	25.24 ± 5.97	24.85 ± 5.54	25.61 ± 6.41	0.628
1 year post-op	46.74 ± 4.71	46.14 ± 4.46	47.30 ± 4.94	0.345
Significance within group		< 0.01	< 0.01	

IMT, induced membrane technique; PCS, physical component summary; MCS, mental component summary

**Table 3:** Bacterial species detected following culture after surgery of 103 patients with atrophic nonunion

Species	Total (*N* = 103)	Patients treated with two-stage IMT (*N* = 41)	Patients treated with one-stage autograft (*N* = 62)	*P* value
Total	17 (16.5)	6 (14.6)	11 (17.7)	0.79
Coagulase-negative Staphylococcus spp.	8 (7.77)	3 (7.32)	5 (8.1)	
Propionibacterium acnes	2 (1.94)	–	2 (3.2)	
Enterococcus faecalis	2 (1.94)	1 (2.44)	1 (1.6)	
Bacillus spp.	2 (1.94)	1 (2.44)	1 (1.6)	
Sporolactobacillus laevolacticus	1 (0.97)	–	1 (1.6)	
Delftia acidovorans	1 (0.97)	–	1 (1.6)	

IMT, induced membrane technique

In this study, 95 (92.2%) patients achieved bone healing at 12 months of follow-up. The bone healing rate was comparable in both groups (IMT: 92.7% vs. one-stage: 91.9%, *P* = 0.089), although the bone healing Lane and Sandhu score was superior in the IMT group (8.68 ± 0.96 vs. 7.81 ± 1.76, *P* = 0.002) (Table 2). Within the total follow-up, 19 (18.4%) patients had complications after treatment, of whom 8 (7.8%) patients had nonunion, 3 (2.9%) patients had infection and 2 (6.9%) patients had material failure. An additional revision to achieve final success was needed among these patients, three of whom received one-stage autologous grafting and ten of whom received two-stage IMT treatment again. In addition, six patients had mild pain at the donor site (anterior iliac) area. For all these complications, there was no significant difference between the two groups (Table 2).

### Quality of life and healthcare burden

Before and after surgery over a minimum of a 1-year follow-up period, patients’ healthy quality of life was longitudinally surveyed. Among all atrophic nonunion patients, the preoperative self-reported SF-12 scores, including physical composite (mean: 22.16 ± 6.84) and mental composite scores (mean: 25.24 ± 5.97), were very poor and were significantly below the total scores. Both the physical composite scores (mean: 21.36 ± 5.96 vs. 49.64 ± 3.75) and mental composite scores (mean: 24.85 ± 5.54 vs. 46.14 ± 4.46) were significantly improved at 12 months after treatment in the IMT groups as well as in the one-stage autologous grafting group, with no significant difference between the two groups (Table 3). In addition, since the staging method of IMT causes an increase in the number of operations, the total hospital stays (median: 8 days vs. 14 days, *P* < 0.01) and direct healthcare costs (¥30,432 vs. ¥56,327, *P* = 0.026) were significantly greater in the IMT group (Table 3).

## Discussion

Atrophic nonunion [1, 9, 17] is mostly caused by inadequate local blood supply and low osteogenic potential, and low toxic infection may also be another important cause. At present, the revision surgery strategy is mainly based on the "diamond theory," which was first proposed by Professor Giannoudis [6]. In the management of nonunion, the author indicated that factors such as peripheral blood vessels, osteoinductive factors, osteoblasts, bone conduction matrix and mechanical stability are most important and need special attention. In this study, all atrophic nonunion patients were treated according to the "diamond theory" [5, 6], and both methods applied in our cohorts aimed to improve biological conditions to achieve final bone healing.

Currently, the treatment options for atrophic nonunion include autologous bone grafts, the use of biological agents and stem cell transplantation. Obviously, the treatment of biological agents [21] and stem cell [22] transplantation are expensive and often have high technical requirements. Recently, Łukasz Szelerski et al. [23, 24] also reported a new method of Ilizarov to treat nonunion, and excellent long-term union was achieved in their cohorts. The greatest benefit of this approach is that there is no need for additional bone grafting, and the damage of the implant to the local blood supply can be reduced. However, the long duration of Ilizarov stabilization (a mean of 7.9 months) in these patients is a concern, and atrophic nonunion also seems to be a risk factor with this method in their reports. Among these, autologous bone grafting [1, 5, 17] is a standard method that provides all the essential properties of bone healing, such as osteoblasts, bone conduction matrix, and bone induction factors. Previously, this standard treatment was often performed within one step (one-stage autologous bone grafting).

However, IMT provides another two-stage treatment strategy [11–15]. In this two-stage surgical method, bone cement is first implanted in the nonunion site after debridement, while bone grafting is performed in the second stage after 6–8 weeks. Many reports [13, 18, 25] have indicated that IMT can significantly improve the success of bone defect treatment compared to one-stage bone grafting in the management of large bone defects. The key to osteogenesis is that an induced membrane [10, 14] can form around the cement; it is highly vascularized and can secrete various growth factors, including transforming growth factor beta 1 (TGF-β1), fibroblast growth factor 2 (FGF-2), bone morphogenetic protein 2 (BMP-2) and vascular endothelial growth factor (VEGF), and it can mobilize precursor cells to promote bone healing. Moreover, PMMA bone cement can also include antibiotics to treat the underlying infection [11, 15]. Comparing the IMT to other surgical procedures, the recent review [26] showed that better functional results were achieved in bone defects due to infection with IMT treatment. Therefore, IMT is theoretically ("diamond theory") more in line with the treatment of atrophic nonunion to improve the biological environment of the fracture site.

In this study, the two-stage method of IMT was used to treat atrophic aseptic nonunion and compared with one-stage autologous bone grafting. Before that, the baseline demographic (e.g. age [1]) and clinical characteristics (e.g. hardware used [27]) were compared, since these can be potential confounding factors affecting the bone union. The cure rate (92.7% vs. 91.9%, *P* = 0.089) and total complications (17.1% vs. 19.4%, *P* = 0.770) were comparable in both groups; however, the final Lane and Sandhu score was superior within the IMT treatment group (8.68 ± 0.96 vs. 7.81 ± 1.76, *P* = 0.002). Previously, Moghaddam [28, 29] et al. reported that the success rate of atrophic nonunion treatment with one-stage autologous bone grafting ranged from 80 to 90%. Our results are supportive of the idea that two-stage IMT serves as an alternative in treating atrophic nonunion since it can achieve good healing by improving the local biological environment.

In addition to the above clinical results, patients’ health quality of life [30, 31] and treatment burden were also assessed in this study. It is clear that the two-stage method of IMT, compared to one-stage autologous bone grafting, significantly increases the number of operations, hospital stays and total medical costs. With respect to patients’ quality of life, we found that both the SF-12 physical composite and mental composite were below the 50th percentile of the total score. These findings were similar in other studies on femoral [32] and tibial [3] nonunions, indicating a detrimental effect on patients’ quality of life. Both the stage method of IMT and one-stage autologous bone grafting can significantly increase patients’ quality of life after surgery; however, no statistically significant difference was found within groups in this study. In comprehensive patient clinical outcomes, quality of life and treatment burden, we suggest that the staging method of IMT seems to not be a preferred option in the management of atrophic nonunion. More evidence-based research and cost-utility analysis are needed to further guide clinical decision-making.

Based on the "diamond theory," the stage method of IMT should be regarded as a supplement to one-stage autologous bone grafting in the management of atrophic nonunion. According to our experience and previous results in the literature, IMT may be more suitable for the following clinical settings: (1) patients with a high risk of infection (e.g. suffering severe open fractures and long-term smoking), as IMT can treat potential low toxicity infections [15]. The results from our total cohort revealed that a proportion of atrophic nonunion patients (*n* = 17, 16.5%) had infections with low toxicity. Another study [9] indicated that as many as 57% of patients were associated with low-grade infection; (2) patients with large bone defects or without enough autologous bone grafts who require the addition of bone substitutes (e.g. allogeneic bone) since IMT [10, 33, 34] can further improve the local biological environment; and (3) patients who failed previous one-stage autologous bone grafting for unknown reasons. Of note, the treatment options should also reflect some sensitivity to patient tolerance, including patient compliance, increased surgery or costs [35] and patient quality of life [36], as emphasized above in this study.

There are some limitations to this study. First, the total number of patients was relatively small. Although nonunion is a serious complication of fracture and the overall population is large, patients with hypertrophic nonunion or infectious nonunion were excluded from our study. Second, our present study is a retrospective study, and incomplete clinical data collection may occur within the retrospective analysis. Finally, patients choosing the different surgical protocols may have bias, although we have been very neutral to surgical information for both methods. Overall, this study provides a comparison of the two-stage induced membrane technique and one-stage autografting in the management of atrophic nonunion, and our findings will provide basic information for patient and clinician decision-making as well as patient consultation.

## Conclusion

The stage method of IMT should be regarded as a supplement to one-stage autologous bone grafting in the management of atrophic nonunion. Current data also indicate that the staging method of IMT may not be a preferred option in comprehensive considering patient clinical outcomes and healthcare burden, although it serves as an alternative in treating atrophic nonunion. The IMT may be more suitable in some high-risk clinical settings by improving the local biological environment. The results of future large-sample, randomized controlled studies and cost-utility analyses are needed to better guide clinical decision-making.

## Data Availability

The datasets used and/or analysed during the current study are available from the corresponding author upon reasonable request.
